# Anesthetic Management of Intra-aortic Balloon Pump-Induced Systolic Anterior Motion of the Mitral Valve During Coronary Artery Bypass Grafting

**DOI:** 10.7759/cureus.56815

**Published:** 2024-03-24

**Authors:** Rachel Figaro, Imani Thornton, Jeremy P Scott, Joseph Sluhoski

**Affiliations:** 1 Anesthesiology, HCA Florida Westside Hospital, Plantation, USA; 2 Anesthesiology and Critical Care, HCA Florida Westside Hospital, Plantation, USA; 3 School of Medicine, Ross University School of Medicine, St. Michael, BRB; 4 Cardiothoracic Anesthesiology, HCA Florida Westside Hospital, Plantation, USA

**Keywords:** left ventricular outflow tract obstruction (lvoto), coronary artery bypass graft (cabg), perioperative tee, cardiothoracic anesthesia, intra-aortic balloon pump (iabp), hypertrophic obstructive cardiomyopathy (hocm), systolic anterior motion of mitral valve

## Abstract

The intra-aortic balloon pump (IABP) is a mechanical device that increases myocardial oxygen perfusion and indirectly increases cardiac output through afterload reduction. Since its inception, the IABP has been a mainstay of cardiac support devices, utilized as a temporizing measure in patients with or prone to developing cardiogenic shock that are awaiting definitive treatment. Systolic anterior motion (SAM) of the mitral valve is a well-described phenomenon that can precipitate hemodynamic collapse by obstructing the left ventricular outflow tract in a subset of patients with cardiac pathology, most notably hypertrophic obstructive cardiomyopathy (HOCM). This report describes the case and anesthetic management of a patient who had an IABP placed for support and later developed SAM and hemodynamic compromise after induction of general anesthesia during a coronary artery bypass surgery.

## Introduction

The intra-aortic balloon pump, first clinically implanted in 1967 [1], is an implantable mechanical circulatory device utilized to support hemodynamics by increasing coronary artery perfusion, decreasing afterload, and maintaining cardiac output. A catheter with a balloon at its tip is inserted into the aorta and positioned distal to the left subclavian artery. The balloon inflates during diastole and deflates during systole and through this mechanism, termed counterpulsation, the intra-aortic balloon pump (IABP) provides significant hemodynamic support to avoid cardiovascular decompensation. The ultimate goals of the IABP are to increase myocardial oxygen supply and decrease cardiac demand. Four main indications for its placement are cardiogenic shock, myocardial infarction, stabilization prior to or after cardiac surgery, and unstable angina [2].

Systolic anterior motion (SAM) of the mitral valve describes abnormal motion of the mitral valve that can result in left ventricular outflow tract obstruction (LVOTO). SAM has typically been reported and seen in hypertrophic obstructive cardiomyopathy (HOCM) pathology. The hypertrophied interventricular septum contributes to the narrowing of the left ventricular outflow tract (LVOT), which results in displacement of the anterior leaflet of the mitral valve into the LVOT during systole. Of the rarer causes of systolic anterior motion of the mitral valve, it can be seen post-myocardial infarction, following surgical mitral valve repair, under general anesthesia, and anaphylaxis [3], to name a few.

In some instances, it has been reported that insertion of the IABP can result in paradoxical hypotension [4]. Here, we evaluate the case of a woman who was admitted to our institution following a myocardial infarction with such unique pathology that she developed SAM of the mitral valve with subsequent hypotension while an IABP was in place during surgery. We will identify her unique predisposition to SAM and investigate how augmentation from the IABP while under general anesthesia further worsened her function, leading her to develop cardiogenic shock. Through this patient’s case, we will explore anesthetic management of IABP-induced SAM during coronary artery bypass graft surgery while acknowledging the importance of prompt recognition of the cause of her hypotension and survey the different diagnostic modalities utilized in such an acute setting.

## Case presentation

We present the case of a 69-year-old female with uncontrolled hypertension, uncontrolled diabetes, and coronary artery disease who has never taken any medications. This patient was transferred to our institution for an acute anteroseptal ST-segment elevation myocardial infarction (STEMI) after a five-day admission in another facility for unremitting chest and back pain. Immediate heart catheterization upon her arrival revealed 99% occlusion in the proximal and mid portions of the left anterior descending artery (LAD) and 70% occlusion in the right coronary artery (RCA). During right coronary angiogram, the patient developed unstable ventricular tachycardia and required immediate cardioversion at 360 Joules, which converted her to sinus rhythm. An IABP was subsequently placed to support hemodynamics, and the patient was transferred to the cardiac intensive care unit (ICU) to await coronary artery bypass graft (CABG) surgery the following day.

On the day of her CABG, the patient was seen in the ICU with her blood pressure ranging from 90-100s systolic and 50-60s diastolic with the IABP at 1:1 augmentation. An arterial line was successfully placed in the right radial artery and the patient was transported to the operating room. General anesthesia was induced with 150 mcg of fentanyl, 20 mg of etomidate, and 100 mg of succinylcholine. The patient was intubated with a 7.5 mm endotracheal tube and placed on positive pressure ventilation, the patient became hypotensive with her blood pressure ranging from 60-70s systolic, 30-40s diastolic, mean arterial pressure in the 40-50s, and heart rate in the 60s with minimal response to phenylephrine and little to no response to epinephrine. Once central access was achieved and a pulmonary artery catheter was positioned with no complication, the following were obtained: continuous cardiac index, measured through advanced thermodilution, was 1.4 L/min, central venous pressure was 16 mmHg, systolic pulmonary artery pressure was 32 mmHg and diastolic pulmonary artery pressure was 19 mmHg. The patient eventually required a norepinephrine infusion at 6 mcg/min to which she responded and remained on for the duration of the procedure.

**Figure 1:**
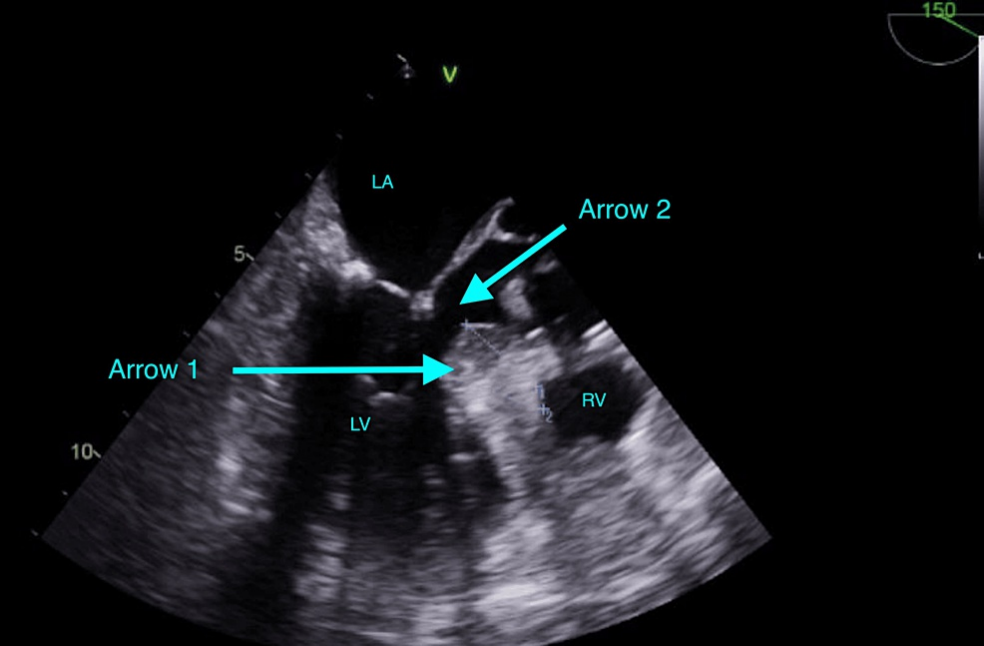
TEE mid-esophageal long axis view demonstrating asymmetric hypertrophy of the basal anteroseptal wall of the left ventricle. Arrow 1 highlights the thickest portion of the septum, measuring 21.7 mm, owing to its sigmoid shape. Arrow 2 points to the left ventricular outflow tract. LA- left atrium, LV- left ventricle, RV- right ventricle

Once the transesophageal echocardiography (TEE) probe was placed, the IABP was visualized and in good position, but the patient had asymmetric hypertrophy of her left ventricular wall and systolic anterior motion of the mitral valve, resulting in impingement of the LVOT. The basal anteroseptal wall of the left ventricle heralded a sigmoid shape and was measured to be 21.7 mm in width (Figure 1), greater than the 15 mm minimum that is traditionally indicative of hypertrophic obstructive cardiomyopathy. The C-sept, which is the shortest distance between the coaptation of the mitral valve at the end of systole and the ventricular septum, was measured as 1.71 cm, thus contributing to her SAM. A C-sept of less than 2.75 cm suggests there is anterior displacement of the mitral valve, and can result in SAM. In addition, the left ventricular ejection fraction was 50%, and there was moderate mitral regurgitation, mild tricuspid regurgitation, and mild pulmonary insufficiency.

The general anesthesia and positive pressure ventilation in conjunction with IABP augmentation at 1:1 was worsening this patient’s hypotension by creating a significant gradient in the LVOT. When the IABP was temporarily turned off to evaluate her response under direct visualization with TEE, the LVOT gradient was reduced and the patient’s hemodynamics rapidly improved. We then continued the procedure with the IABP at 1:3 to minimize the decrease in afterload as the afterload needs to be maintained when managing patients with SAM and HOCM.

Throughout the CABG, the goals for the management of this patient’s anesthesia were a combination of increased volume to support preload, decreased contractility to provide adequate filling time, and increased afterload to maintain hemodynamics. The early recognition of the asymmetric left ventricular hypertrophy and systolic anterior motion of the mitral valve allowed for the patient to be hemodynamically optimized. Once her unique pathology, secondary to HOCM, which was exacerbated by the IABP was detected, the anesthesiology and surgical teams agreed to have the IABP remain at 1:3 until the patient was placed on cardiopulmonary bypass. While weaning off bypass, attempts had been made to reinstitute the IABP at 1:1, however, the patient’s hemodynamics did not tolerate its use, and she was maintained with vasopressors. She was successfully weaned from cardiac bypass and required infusions of norepinephrine at 2 mcg/min, vasopressin at 0.04 U/min, and milrinone at 0.25 mcg/kg/min to aid in offsetting the rising peak pulmonary artery pressures. Prior to transferring the patient to the cardiovascular ICU following her CABG, the IABP was removed as risks of leaving it in place such as migration when transporting the patient and limb ischemia, to name a few, outweighed the benefits that were achieved with pharmacologic support.

This case provides an example of systolic anterior motion of the mitral valve induced by an intra-aortic balloon pump. The induction of general anesthesia resulted in vasodilation with a reduction in preload and the addition of inotropes increased cardiac contractility that worsened her hypotension. The IABP decreased her much-needed afterload and contributed to a worsened LVOTO. Once the IABP was momentarily turned off and left at 1:3, the hypotension improved significantly, and TEE revealed the resolution of the left ventricular outflow tract obstruction.

## Discussion

Systolic anterior motion of the mitral valve is an occurrence that has traditionally been a pathognomonic feature for hypertrophic obstructive cardiomyopathy, first described in the 1960s [5]. However, several other cases throughout the literature have identified the occurrence of SAM of the mitral valve in the absence of HCM. For example, a study evaluating outcomes after mitral valve repair identified the incidence of SAM after surgical mitral valve annuloplasty to be 6.6%, down from the 10% from 10 years prior [6, 7]. Other studies report dynamic left ventricular outflow tract obstruction following acute myocardial infarction with the mechanism theorized to be from compensatory ventricular hyperkinesis [8]. Other authors have identified SAM to occur in conjunction with surgical aortic valve repairs, comorbid conditions such as hypertension and diabetes, and in cases of significant hypovolemia or anaphylaxis [3]. We hypothesize that our patient had a higher risk of developing systolic anterior motion of the mitral valve and subsequent hypotension secondary to her unique confluence of comorbidities and events: uncontrolled hypertension leading to left ventricular hypertrophy with the left ventricular septal wall predominantly affected, diabetes, acute myocardial infarction, the induction of general anesthesia, and most notably, the presence of the intra-aortic balloon pump.

It has been established that the IABP is generally placed to support hemodynamics. With ideal placement of the balloon tip just distal to the subclavian artery, it inflates during diastole to improve coronary blood flow and aids in displacing blood from the aorta to the periphery. The balloon then rapidly deflates before the onset of systole and, in doing so, decreases afterload, reduces cardiac work and myocardial oxygen demand. A study evaluating the aortic function of patients with cardiogenic shock before and during intra-aortic balloon pumping found that aortic distensibility improved, resulting in a 24% increase in cardiac index and a 31% decrease in myocardial oxygen demand [9]. In addition, a Cochrane review in 2011 found that placement of IABPs prior to CABGs may be related to reduced morbidity and mortality and decreased duration on cardiopulmonary bypass [10].

Through this case, we witnessed how the use of an IABP in conjunction with general anesthesia and our patient’s unique comorbidities resulted in cardiovascular collapse that necessitated the use of vasopressors but essentially required discontinuation of the IABP. It was evident that a multitude of factors contributed to this hypotension: the decreased systemic vascular resistance from the anesthetics, the reduction in afterload largely imparted by the IABP, the reduction in preload from general anesthesia and her relative hypovolemia from fasting, and the use of inotropes that resulted in a hypercontractile left ventricle. A slight resolution of her hypotension was seen with increased fluid and a lower inhaled anesthetic concentration with maintaining her bispectral index (BIS) at 40-60. However, it was peculiar that these adjustments did not result in significant improvement in the LVOTO and hemodynamics until the IABP was set to 1:3.

Regarding the IABP, many cases explain how it can lead to altered hemodynamics with the inflation and deflation of the balloon [4]. In addition, a well-described Venturi effect can occur from an increased pressure gradient developing after balloon inflation with a resultant partial obstruction of aortic outflow and acceleration of blood flow. This acceleration causes a left ventricular suctioning force, leading to the anterior mitral valve leaflet creating a left ventricular outflow tract obstruction. This, combined with a high-contractile force generated by the left ventricle during systole creating a higher-pressure environment further predisposes the anterior mitral valve leaflet to move anteriorly and contribute to SAM and LVOT obstruction.

**Figure 2:**
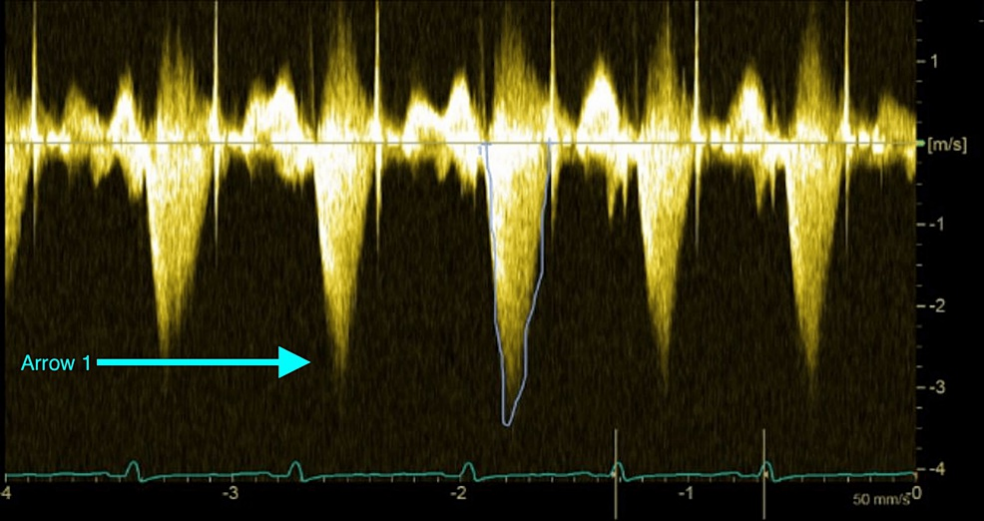
TEE image of continuous wave Doppler through the LVOT while the IABP was at 1:1 counterpulsation. This waveform demonstrates the characteristic “dagger shape” morphology of left ventricular outflow tract obstruction. Arrow 1 highlights the late systolic peak. TEE: transesophageal echocardiography; LVOT: left ventricular outflow tract; IABP: intra-aortic balloon pump

**Figure 3:**
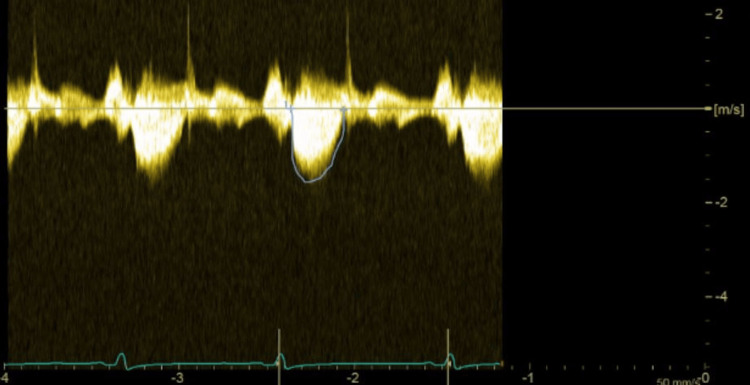
TEE image of continuous wave Doppler with no significant obstruction through the LVOT with the IABP at 1:3 counterpulsation. TEE: transesophageal echocardiography; LVOT: left ventricular outflow tract; IABP: intra-aortic balloon pump

Intraoperative TEE was paramount in quickly recognizing that the SAM and LVOT obstruction were primarily induced by the intra-aortic balloon pump. When the IABP was at 1:1 counterpulsation, the characteristic “dagger shape” waveform with a late systolic peak in the continuous spectral Doppler signal through the LVOT was observed, which is diagnostic of a dynamic left ventricular outflow tract obstruction (Figure 2). This wave morphology was present in our patient because ejection at the beginning of systole is generally normal but as the pressure increases, the left ventricular outflow tract continues to narrow, resulting in a dynamic obstruction. When measured, the maximum LVOT pressure gradient was approximately 48 mmHg, suggestive of severe LVOT obstruction. In comparison, when the intra-aortic balloon pump was briefly turned off, the waveforms returned to their normal, elliptical shapes (Figure 3), the maximum LVOT gradient decreased to 27 mmHg, and the patient’s hemodynamics started to improve, leading to the decision of maintaining the IABP in 1:3. Based on all these factors, it is safe to conclude that the systolic anterior motion of the mitral valve and left ventricular outflow tract obstruction were secondary to the intra-aortic balloon pump while the patient was under general anesthesia.

The anesthetic management of this patient was a difficult task as the IABP that was placed to support hemodynamics exacerbated the sequelae of this patient’s left ventricular hypertrophy and resulted in SAM. TEE proved beneficial in diagnosing her pathology and making decisions regarding her management.

## Conclusions

Although our patient was uniquely predisposed to developing systolic anterior motion of the mitral valve, the use of the intra-aortic balloon pump under general anesthesia made her prone to hemodynamic compromise. In conjunction with the recent myocardial infarction and induction of general anesthesia, the intra-aortic balloon pump created the perfect situation for the development of SAM and the LVOT obstruction that resulted in her hypotension. Prompt insertion of the transesophageal echocardiography probe allowed for the immediate recognition of SAM and the implementation of the proper anesthetic management to further achieve hemodynamic stability and guide her progress throughout the CABG procedure. Setting the IABP to 1:3 counterpulsation, maintaining adequate preload, avoiding hypercontractility, and increasing systemic vascular resistance to reduce the left ventricular outflow tract gradient were paramount in this patient’s anesthetic management.
